# Correction to: labelSeg: segment annotation for tumor copy number alteration profiles

**DOI:** 10.1093/bib/bbae073

**Published:** 2024-02-17

The online **Article history** should read:

“**Received**: 31 August 2023

**Revision received**: 09 November 2023

**Accepted**: 28 December 2023

**Published**: 31 January 2024”

instead of:

“**Received**: 31 August 2023

**Revision received**: 09 November 2023

**Published**: 31 January 2024

**Accepted**: 28 December 2024”.

Within the PDF, these details should read:

“**Received**: August 31, 2023. **Revised**: November 9, 2023. **Accepted**: December 28, 2023” instead of: “**Received**: August 31, 2023. **Revised**: November 9, 2023. **Accepted**: December 28, 2024”.

The publisher apologises for the error. The emendations have been made within the article.

